# Comparison between five coprological methods for the diagnosis of *Balantidium coli* cysts in fecal samples from pigs

**DOI:** 10.14202/vetworld.2021.873-877

**Published:** 2021-04-12

**Authors:** Juan Carlos Pinilla, Andrea Isabel Pinilla, Angel Alberto Florez

**Affiliations:** 1 Department of Veterinary Medicine, Faculty of Exact, Natural and Agricultural Sciences, University of Santander, Bucaramanga, Colombia; 2 Department of Microbiology, Faculty of Health, Industrial University of Santander, Bucaramanga, Colombia

**Keywords:** *Balantidium*, parasite, pigs, protozoan

## Abstract

**Background and Aim:**

*Balantidium coli* is a protozoan that can infect humans and non-human primates, being the domestic pigs the animals most affected by this parasite. This study aimed to compare the performance of five coprological methods for diagnosis of *Balantidium coli* cysts in fecal samples from pigs.

**Materials and Methods:**

From September to December 2019, 558 fecal samples were collected from the rectum of backyard pigs in the Bucaramanga metropolitan area, Colombia. The average age of the sampled animals was 3-4 months. Fecal samples collected were tested using the following coprological techniques: Direct examination with Lugol’s iodine solution, buffered saline, centrifugation/flotation and McMaster techniques, and Ziehl–Neelsen method.

**Results:**

The results indicate that *B. coli* cysts were diagnosed most frequently through direct examination with Lugol’s iodine solution (52.7% of the samples) followed by direct examination with a buffered saline solution (37.6%). Moderate concordance (k=0.41; p<0.05) was determined between direct method with Lugol’s iodine and buffered saline solution, McMaster and buffered saline (k=0.35; p<0.05), and centrifugation/flotation and buffered saline (k=0.28; p*<*0.05) showed a fair degree of concordance. The rest of the comparisons were classified as poor. The flotation techniques (centrifugation/flotation and McMaster) did not show good recovery of cysts.

**Conclusion:**

It is concluded that the most efficient method for diagnosing this parasitosis was to an association between direct examination with Lugol’s iodine solution and buffered saline.

## Introduction

*Balantidium coli* is a ciliated protozoan that can infect humans and non-human primates; however, domestic pigs are the animals most affected by this parasite. This protozoan has a cosmopolitan distribution with prevalence ranges in pigs between 33% and 94% [1]. In humans, balantidiosis can manifest clinically as asymptomatic hosts that can serve as reservoirs of infection to patients with severe balantidiosis passing mucoid, bloody stools [2,3]. This parasitic disease is acquired by humans through the fecal–oral route from the pig which is the normal asymptomatic host [4]. This protozoan has two evolutionary forms: Trophozoite and the cyst. The trophozoite can measure up to 120 μm in width by 150 μm in length, while the cyst, which may be spherical, measures 40-60 μm in diameter [4].

The diagnosis of *B. coli* is based on the observation of evolutionary forms in feces, by light optical microscopy [4]. Due to its large size and spiral motility, *B. coli* trophozoite can be easily recognized on wet slide preparations; the cyst, by its spherical form is also easily recognizable [4].

Several authors have reported the use of different coprological techniques for the diagnosis of *B. coli*. Nishi *et al*. [5] used flotation methods with glucose for the diagnosis of protozoan in pigs. Weng *et al*. [6] used direct methods and flotation solution with sodium chloride for the diagnosis. On the other hand, Sanchez [7] using direct examination for diagnosing *B. coli* in human stool samples, and Barbosa *et al*. [8] compared five coprological techniques for the diagnosis of *B. coli* cysts in fecal samples from pigs and non-human primates. Azevedo *et al*. [9] used four parasitological techniques to study intestinal parasites in fecal samples from people from Brazil.

The clinical diagnosis of intestinal parasitic infections is inaccurate because it is based on non-specific clinical manifestations. Therefore, laboratory diagnosis plays an important role in the diagnosis of these infections [10]. Despite the existence of numerous quantitative and qualitative techniques for parasitological examination of feces, there is very little information about studies that have evaluated the performance of diagnostic methods to identify *B. coli* cysts [8]. A reference technique with a high percentage of precision and security is not generally available for parasitological studies, especially to detecting gastrointestinal parasites [11].

Therefore, it is important to review coprological diagnostic methods to determine more accurate results for the diagnosis of this parasitosis. Accordingly, the aim of this study was to compare five coprological techniques for laboratory diagnosis of *B. coli* cysts in fecal samples from pigs.

## Materials and Methods

### Ethical approval

This research was approved by the Institutional Ethical Committee of the University of Santander (ref No CIF0311-19).

### Study site and period

The research was conducted in farms located in the Bucaramanga metropolitan area (7°08′00″N 73°08′00″W), department of Santander, Colombia [12]. This region comprises a geographical area of 1479 km^2^. Rainfall is regular throughout the year; however, more rains are experienced from October to December. Bioclimatic characteristics of the region are similar and with the mean annual temperature of 25°C, with little weather varies throughout the year. Altitude is between 600 and 1700 masl and the mean annual rainfall is 1040 μm, with 78% relative humidity [12]. The study was conducted from September to December 2019.

### Study design

A cross-sectional and descriptive study was designed. Sixty-four backyard pig farms were visited, and fecal samples were collected weekly. Most of the pigs sampled were crossbreeds between Yorkshire, Landrace, and Pietrain. The average age of the sampled animals was 3-4 months. The expected sample size was determined using the formula provided by Thrusfield [13]. As the prevalence of *B. coli* in the local pig population was unknown, the hypothesized prevalence of 75%, with a confidence level of 95%, an estimated sample size of 558 fecal samples was determined to compare the five coprological diagnostic methods. Eight to nine fecal samples were randomly collected from each farm examined. A sample was considered positive if at least it showed one protozoan cyst.

### Sample collection and laboratory analysis

Fecal samples were collected from the rectum of pigs using palpation gloves. Approximately 10 g of feces without chemical preservative were obtained from each animal. Samples were placed into containers filled with ice packs and immediately transported to the laboratory for processing. Part of the fecal sample was processed by direct methods with Lugol’s iodine solution (1:5 dilutions) and buffered saline [14], and the rest of the sample was processed by the other coprological techniques. For the McMaster technique, 2 g of feces were mixed with 28 mL of the sugar-salt flotation solution (1 L of saturated NaCl plus 500 g of sugar, at room temperature (27°C) and 1.32 g/mL specific gravity) [15], in a 50 mL Falcon tube, and vigorously agitated until complete homogenization and left undisturbed. After 6-8 min, a 0.5 mL sample of the supernatant was taken and placed in the McMaster chamber for reading. *B. coli* cysts were counted, and the total number of cysts was multiplied by a coefficient of 50 [16]. However, a sample was considered positive if at least one cyst was observed in the chambers. The centrifugation/flotation technique (Sheater technique) included 2-3 g of feces that were suspended in tap water and sieved (mesh size: 250 μm) into a 100 mL glass beaker. After 3 min of sedimentation, the supernatant was discarded, and the sediment was transferred to a 10 mL tube and centrifuged (400 g × 5 min). The supernatant was discarded again, and the sediment was resuspended in a tube with sugar-salt solution up to a formation of the convex meniscus. After 10 min of flotation, the meniscus was lifted with a microscopical slide and examined microscopically to 100× [17]. For the Ziehl–Neelsen method, a thin layer of feces was transferred to the center of the slide and spread the material with a cotton swab. Air-dried at room temperature (27°C), fixed with methanol for 5 min and colored with carbol fuchsin for 4 min, and rinse until the water runs clear. Then, wash and decolorized with the hydrochloric acid-ethanol and rinse until the water is clear. Cover the slide with methylene blue for 1 min, wash with tap water and dry. The slide was observed microscopically with immersion oil at 40× and 100×. The cyst structure, if present, is seen in purple red on a blue background [14].

### Statistical analysis

The results of this study were analyzed by the Chi-square test to determine statistical differences, with confidence intervals of 95%. The kappa index (k) was calculated to determine the concordance among the five coprological technics, and this kappa index was interpreted according to the classification indicated by Barbosa *et al*. [8]. The level of significance for the analyses was 5%. Calculations were made using the SPSS Statistics for Windows (IBM, USA), version 21.0 [18].

## Results

*B. coli* cysts were detected in 79.2% of the fecal samples, by at least one coprological technique. Table-1 shows the comparison (Chi-square test) between recovery rates of *B. coli* cysts among the five coprological techniques used. Different recovery percentages were observed, suggesting a statistical association (p<0.05) with respect to the technique. *B. coli* cysts were observed most frequently using the direct method with Lugol’s iodine solution (52.7%), followed by the direct examination with a buffered saline solution (37.6%), centrifugation/flotation technique (29.7%), and McMaster technique (19%). The Ziehl–Neelsen method showed poor performance in detecting *B. coli* cysts (0.35%).

**Table-1:** Recovery rate of *B. coli* cysts by the coprological techniques used (n=558).

Technique	Positives	Negatives	%	p-value
Lugol’s iodine solution	294	264	52.7	0.000
Buffered saline solution	210	348	37.6	
Centrifugation/flotation	166	392	29.7	
McMaster	106	452	19	
Ziehl–Neelsen	2	556	0.35	

Statistically significant (p<0.05). *B. coli=Balantidium coli*

Regarding the kappa index, the highest values were observed in the comparison between direct examination with Lugol’s iodine and buffered saline solution (p<0.05), showing a moderate concordance between both methods (k=0.41; p<0.05). On the other hand, the comparisons between McMaster and buffered saline (k=0.35; p<0.05) and centrifugation/flotation and buffered saline (k=0.28; p*<*0.05) showed a fair degree of concordance. The rest of the comparisons were classified as poor, and the kappa index was not statistically significant (p>0.05) (Table-2).

**Table-2:** Concordance of the results obtained through coprological techniques applied to *B. coli* cysts (n=558).

Technique	k	p-value
Lugol×BS	0.41	0.000
MM×BS	0.35	0.005
CF×BS	0.28	0.000
MM×CF	0.03	0.5
BS×ZN	−0.1	0.34
Lugol×ZN	−0.02	0.5
CF×ZN	−0.02	0.58
MM×ZN	−0.03	0.49
Lugol×CF	−0.05	0.9
Lugol×MM	−0.005	0.23

*B. coli=Balantidium coli*, k=Kappa index, CF=Centrifugation/flotation, MM=McMaster, ZN=Ziehl–Neelsen, BS=Buffered saline. Statistically significant (p<0.05)

## Discussion

**Figure-1:**
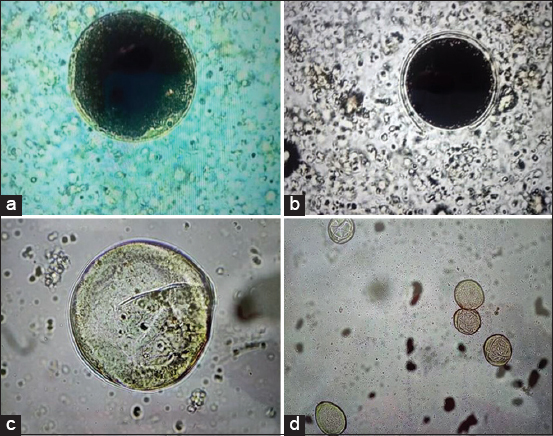
Cysts of *Balantidium coli* observed in fecal samples from pigs by different coprological techniques: (a): Lugol’s iodine solution, (b): Buffered saline solution, (c): Centrifugation/flotation technic, (d): McMaster technic.

*B. coli* is a protozoan with a zoonotic potential worldwide. Most infections by this pathogen in pigs are subclinical and usually limited to the small intestine. In some cases, after injures of the intestinal wall by other agents as bacteria and virus, the trophozoites can penetrate the mucosa causing diarrhea, bloody feces, and even death of the pig. Therefore, the diagnosis of this protozoan must be opportune [19]. In this research, the use of the five coprological methods allowed detecting *B. coli* cysts, in the fecal samples from pigs. However, other studies on the examination of *B. coli* have reported using at least two coprological methods [5,6]. Overall, direct examination with Lugol’s iodine solution presented the highest recovery rate of detection of *B. coli* oocysts followed by examination direct with a buffered saline solution. Probably, *B. coli* cysts tend to be found more on the superficial layer of the fecal sample, since the material from digested food goes for a few movements, therefore hindering the spread of parasitic structures. Therefore, fecal scraping may have favored the observation of the protozoan as indicated by Barbosa *et al*. [8]. According to Figueroa-Castillo *et al*. [14], Lugol’s iodine is a rapid, non-specific contrast dye that is added to direct wet mounts of fecal material to aid in differentiating parasitic cysts from other non-parasitic structures. In this sense, many protozoa as *B. coli cysts* (Figure-1a) absorb the dye and appear brown while other objects in the sample remain clear. On the other hand, the buffered solution did not stain the cysts, which often made it difficult to observe them (Figure-1b) in the light microscope. The direct methods have many advantages, especially its methodological facility, short runtime, and low cost; therefore, it should be recommended in the parasitological routine for the recovering of protozoan cysts and trophozoite in fecal samples without chemical preservatives [20]. According to Dryden *et al*. [21], flotation methods are the most widely used in veterinary parasitology. These technics are frequently used to recover lightweight parasitic such as protozoan cysts and oocysts [21]. However, in our study, the lowest values of cysts recovery were determined employing the centrifugation/flotation (29.7%) and McMaster (19%) methods. Although in these two methods, the sugar-salt solution makes it difficult for fecal detritus to float, which facilitates the viewing of parasite structures [22], in this study, *B. coli* cysts tended to become wrinkled through losing liquid to the more concentrated medium (Figure-1c and d), even if the microscope slide was read immediately after laboratory analysis. This alteration was detected in the present study, thus indicating that the sugar-salt solution used in flotation methods generally causes deformities in *B. coli* cysts. Therefore, these technics showed lower performance for the diagnosis because it gave rise to morphological changes to the cysts, causing often it difficult to recognize them. This morphological alteration detected had already been indicated by Dryden *et al*. [21], as a problem. According to these authors, sugar solutions could compromise the integrity of protozoan cysts, being a problem in diagnosing of these endoparasites. On the other hand, the McMaster technique is a coprological method that utilizes fecal samples to determine the fecal egg count, moreover, permitting the detection of parasitic elements as helminth eggs, protozoan oocysts, and cysts [23,24]. This technique is widely used by veterinarians and plays a crucial role in monitoring parasite charges in herds and in epidemiological studies [25]. However, in our study, this technique showed low efficiency in the diagnosis because this technique could be affected by a low amount of stool (2 g), higher multiplication factor (50), and the absence of centrifugation. According to Roepstorff and Nansen [24] and Vadlejch *et al*. [25], this technique improves the sensitivity when enough amount of fecal material (4 g), a low multiplication factor (20), and centrifugation steps are selected. We think that with the lack of centrifugation, the fecal suspension was not sufficiently clear for the examination of *B. coli* cysts. In our study, we have used the Ziehl–Neelsen technique to visualize *Cryptosporidium* oocysts; however, we have also used this technique to find *B. coli* cysts, without good results. A rounded structure of 60 m in diameter with no stain red color was found in all microscopic slides examined. We think that due to its morphological characteristics, it is *B. coli* cyst. This coprological technique is used for the diagnosis of resistant alcohol-acid bacteria like *Mycobacterium* and some intestinal coccidia such as *Cryptosporidium*, *Cyclospora*, *Sarcocystis*, and *Isospora* [14]. These infectious agents have fatty acids in their cell walls that give them the property of resisting decolorization with acid alcohol after using phenolic fuchsin (red color). Therefore, the structure is stained red, allowing its visualization [14]. Probably, the absence of cell wall in *B. coli* cysts does not allow the cyst to stain red when reacting with carbol fuchsine.

When comparing the results obtained by the five coprological methods, not all the kappa index values were statistically significant. In this sense, it was determined that the highest kappa values were observed between direct examination with Lugol’s iodine solution and buffered saline, that is, the methods that showed the highest frequency of positivity. Therefore, these results indicate that the degree of agreement between both methods was moderate by the kappa index. Although this concordance was moderate (k=0.41), it is demonstrated that not all the positive samples were recovered using either technique. Therefore, to increase the performance of detecting *B. coli* cysts, the results of this study show that there is a need to combine both direct methods. The different performance observed in the recovery of *B. coli* cysts from fecal samples from pigs could be associated with the consistency of the sample and the parasitic infection intensity. Moreover, the amount of fecal material examined and the sugar-salt solution used in the coprological analysis [26].

## Conclusion

It is concluded that *B. coli* cysts were more frequently observed by direct examination with Lugol’s iodine solution, and the most efficient method for diagnosing this parasitosis was to an association between direct examination with Lugol’s iodine solution and buffered saline. In this study, the flotation methods were not shown good recovery rates for *B. coli* cysts and therefore are not suitable for investigating this protozoon.

## Authors’ Contributions

JCP conceived and designed the research. JCP and AAF conducted the sample collection. JCP and AIP processed samples in the Laboratory of Veterinary Clinical Research. JCP and AAF carried out the data analysis and writing of the manuscript. All the authors read and approved the submitted version of the manuscript.
